# Editorial: Advances in stem cell engineering: paving the way for regenerative medicine

**DOI:** 10.3389/fbioe.2026.1867976

**Published:** 2026-05-14

**Authors:** Katiucia Batista Silva Paiva, Shue Wang, Sukhbir Kaur

**Affiliations:** 1 Laboratory of Extracellular Matrix Biology and Cellular Interaction, Department of Anatomy, Institute of Biomedical Sciences, University of São Paulo, São Paulo, Brazil; 2 Department of Chemistry, Chemical and Biomedical Engineering, Tagliatela College of Engineering, University of New Haven, West Haven, CT, United States; 3 Laboratory of Pathology, Center for Cancer Research, National Cancer Institute, National Institutes of Health, Bethesda, MD, United States

**Keywords:** 3D bioprinting, cartilage, extracellular vesicles (EVs), hydrogels, instructive microenvironment, mechanotransduction, stem cells, three-dimensional (3D) cell culture models

## Introduction

This Research Topic highlights significant advancements in stem cell biology and regenerative medicine, focusing on how engineered microenvironments and physical cues influence cell behaviour. The studies explore the critical interplay between biochemical signals and biophysical forces, such as mechanical confinement, fluid shear stress, and tissue-specific stiffness, in regulating stem cell differentiation and morphogenesis. By utilising innovative tools such as tunable hydrogels, 3D spheroid cultures, and extracellular vesicles (EVs), researchers are developing strategies to overcome current limitations in cell manufacturing, including cellular senescence and delivery precision. These efforts collectively aim to bridge the gap between laboratory innovation and effective clinical therapies for conditions ranging from cartilage injury to bone remodelling disorders.

Alsehli et al. investigated how physical confinement imposed by synthetic microenvironments affects the differentiation of human induced pluripotent stem cells (hiPSCs) in 3D spheroid models. Researchers observed that under free culture (suspension) conditions, BMP-4 treatment induces axial elongation of spheroids and differentiation into all three germ layers. However, when cells are physically confined in non-degradable Poly(ethylene Glycol)-PEG hydrogels, morphogenesis is hindered and the expression of differentiation markers, such as SOX17, is drastically reduced. The study demonstrates that differentiation can be rescued using hydrogels that soften over time, suggesting that shape change (morphogenesis) is an essential component for the correct specification of cell fate.

Caron et al. explored the mechanosensitive role of the long non-coding RNA (lncRNA) MALAT1 in regulating mesenchymal stem cell (MSC) differentiation under fluid shear stress. The research reveals that physiological levels of shear stress inhibit adipocyte differentiation (fat formation) by reducing lipid droplet accumulation in cells. Using a gapmer double-stranded locked nucleic acid nanobiosensor, the study showed that shear stress downregulated MALAT1, linking an externally applied biomechanical cue to RNA-mediated control of cell fate. Direct silencing of MALAT1 also reduced adipocyte differentiation, confirming that this lncRNA is a key mediator of MSC responses to the mechanical environment of the bone marrow, a crucial factor in understanding disorders such as osteoporosis. More broadly, the work points to lncRNAs as actionable mediators through which engineered physical environments may be translated into lineage-specific outcomes.

One of the translational barriers to the utilisation of stem cells in regenerative medicine or cell therapy is manufacturing. Even when a cell population has promising therapeutic properties, large-scale expansion can alter phenotype, increase senescence, and compromise biodistribution. Pan et al. tackled this problem in placenta-derived MSCs by alternating conventional 2D expansion with transient 3D spheroid culture. This strategy reduced cell enlargement, delayed senescence, preserved proliferative capacity, and enhanced immunomodulatory activity relative to prolonged 2D culture. Importantly, the authors extended the concept toward scalable production by developing RGD-functionalized alginate hydrogel tubes (AlgTubes), enabling repeated transitions between adherent and spheroid states in a continuous format. This contribution reframes culture architecture as a quality-control variable in cell manufacturing rather than a laboratory convenience (Pan et al.).

Yang et al. focused on the potential of mesenchymal stem cells (MSCs) and their extracellular vesicles (EVs) for treating cartilage lesions, which have limited natural repair capacity. Although MSC transplantation is promising, evidence indicates that its regenerative benefits derive mainly from the secretion of EVs rich in bioactive molecules. These vesicles promote chondrocyte proliferation, inhibit apoptosis, modulate local inflammation (polarisation of M1 to M2 macrophages), and assist in the synthesis of the extracellular matrix. The review discusses various sources of extraction (bone marrow, adipose tissue, umbilical cord) and administration methods, including intra-articular injections and hydrogel carriers.

Cruz-Gonzalez et al. proposed a paradigm shift in regenerative medicine through a “bottom-up” approach to biomaterial design in which the biological and microenvironmental requirements of stem cells are identified first, and material properties are engineered around those requirements Instead of adapting cells to existing materials, the strategy first focuses on understanding the biological and microenvironmental needs of stem cells (iPSCs, MSCs, ESCs) and then on creating instructive platforms. The review details advances in 3D bioprinting, extracellular matrix (ECM) scaffolds, and the use of “backpack molecules” to improve the precision of cell therapy delivery. The goal was to overcome challenges such as variability in differentiation and low cell survival after transplantation, transforming passive scaffolds into dynamic environments that promote the functional maturity of derived tissues.

The collective evidence from these studies demonstrates that the future of regenerative medicine lies in the precise control of the cellular microenvironment. This Research Topic highlights the importance of dynamic physical cues—including confinement, stiffness, softening, shear stress, and geometry—in directing differentiation and maturation, as well as molecular indicators such as lncRNA expression, senescence markers, immunomodulatory mediators, and extracellular vesicle cargo. The findings also emphasise the need to incorporate scalability and standardisation early in development, using platforms such as alternating 2D/3D culture systems and biomaterial-assisted delivery strategies to support reproducible translation. Looking forward, precision regenerative medicine will require dynamic biomaterials, physiologically relevant disease models, integrated molecular engineering, and consensus metrics for potency, vesicle identity, senescence, biodistribution, matrix formation, and functional repair.

